# The transient effect of a peer support intervention to improve adherence among adolescents and young adults failing antiretroviral therapy in Harare, Zimbabwe: a randomized control trial

**DOI:** 10.1186/s12981-021-00356-w

**Published:** 2021-06-16

**Authors:** Chiratidzo E. Ndhlovu, Vinie Kouamou, Primrose Nyamayaro, Leanne Dougherty, Nicola Willis, Bisola O. Ojikutu, A. Tariro Makadzange

**Affiliations:** 1 Department of Medicine, University of Zimbabwe College of Health Sciences, Harare, Zimbabwe; 2 John Snow Inc, Boston, MA USA; 3 Africaid, Harare, Zimbabwe; 4 Division of Global Health Equity, Brigham and Women’s Hospital, Boston, MA USA; 5 Ragon Institute of MGH, MIT and Harvard, Cambridge, MA USA; 6 Department of Psychiatry, University of Zimbabwe College of Health Sciences, Harare, Zimbabwe

**Keywords:** HIV, Public health, Adolescents, Sub-Saharan Africa, Zimbabwe, Peer support

## Abstract

**Background:**

Adolescents and young adults living with HIV in sub Saharan Africa are at high risk of poor adherence to antiretroviral therapy (ART) and virologic failure (VF).

**Methods:**

We conducted a randomized control trial among adolescents and young adults on ART with VF to assess the effectiveness of a community-based peer support intervention aimed at improving VF. Viral load (VL) levels were obtained at 12, 24 and 36 weeks. A subset of the participants had baseline HIV drug resistance (HIVDR) genotyped using Sanger sequencing.

**Results:**

The participants’ median (interquartile range (IQR)) age was 18.1 (IQR: 15.1–20.0) years and half (50.5%, n = 107) were male. At week 24, the proportion of subjects with a detectable viremia was significantly lower in the intervention arm than in the standard of care (SOC) arm (76.0% (n = 79) vs. 89.0% (n = 96), p = 0.013). At Week 36, there remained a difference in the proportion of subjects with a detectable VL between the intervention arm (68.3%, n = 71) and SOC arm (79.6%, n = 86), which was trending towards statistical significance (p = 0.059). There was no difference in the probability of having a detectable VL over time between the intervention and SOC groups (adjusted odds ratio: 1.14, p = 0.439). Baseline HIVDR was observed in 44.0% of the participants in the intervention and 56.0% in the SOC group (p = 0.146).

**Conclusion:**

A transient effect of the peer support intervention in improving VF was observed among adolescents and young people failing ART.

*Trial registration*: This study is registered with www.clinicaltrials.gov under the reference number: NCT02833441

**Supplementary Information:**

The online version contains supplementary material available at 10.1186/s12981-021-00356-w.

## Introduction

Since the launch of the ambitious global 90-90-90 targets in 2014 [1], there has been significant progress across the HIV treatment cascade. At the end of 2019, increased access to antiretroviral therapy (ART) had averted an estimated 12.1 million AIDS-related deaths since 2010 [2]. However, the successes were unequal. While ART has been highly effective in reducing AIDS-related deaths among adults, benefits among younger age groups have been limited [3]. Adolescents (10–19 years) have higher rates of attrition, treatment failure, morbidity and mortality compared with adults and children [4–7]. Although the number of deaths is now declining among younger adolescents, deaths among older adolescents have remained unchanged since 2012 [8]. Among young adults (20*–*24 years), high rates of loss to follow-up and poor adherence compared to other individuals living with HIV in sub Saharan Africa have been noted [9–11].

Adolescents living with HIV are a heterogenous group with multiple, complex clinical and psychosocial factors impacting on adherence to ART and retention in care. Among adolescents with perinatally/postnatally acquired HIV infection, delayed ART initiation, chronic infection and co-morbidities have contributed to morbidity and mortality in this age group [8]. Among those acquiring HIV as adolescents, psychosocial and economic challenges complicate ART adherence and engagement in services. Adolescents living with HIV are at increased risk of poor mental health, correlating with poor adherence to ART [12–14].

Evidence of poor outcomes among adolescents living with HIV has led to the development of global and national policies and guidelines to support service delivery for this population [3]. However, globally, there was limited published data describing the efficacy of these interventions in improving adherence and clinical outcomes. Africaid’s Zvandiri (“As I am”) programme is a model of differentiated service delivery for children, adolescents and young people living with HIV [15]. Zvandiri integrates peer-led, community interventions within national HIV service delivery to improve clinical and psychosocial outcomes. Operations research and program data reveal improved adherence, retention, viral suppression and mental health among adolescents receiving Zvandiri services compared with those receiving standard care alone [16–18]. This model has since been documented widely and recommended for scale up [19–21]. Zimbabwe’s Ministry of Health and Child Care has adopted and scaled-up the intervention to 52 (of 63) districts nationally with support from multiple funding partners [22] and the approach has been adopted or adapted in eight countries in the region.

In 2015, at Parirenyatwa Hospital Family Care Clinic (PHFCC) in Harare, adolescent HIV services were provided solely within the clinic which is housed in a tertiary level hospital. Presumed perinatally infected adolescents attending this tertiary level HIV clinic were noted to have delayed linkage to care, with over 50% presenting with advanced disease i.e. WHO stages 3 and 4 [23]. The same study found an early attrition rate of 33% within the first 3 months of enrollment and loss to follow up of 39% at 12 months, and 55% at 60 months [23]. Virologic failure (VF) rates were high among older adolescents (40% among 15–19 year olds). The Zvandiri peer-based intervention demonstrated success in improving clinical and psychosocial outcomes among HIV positive children, adolescents, and youth. However, the approach had not yet been evaluated within a context of youth with high rates of VF. We therefore investigated the effectiveness of this peer support intervention within the context of routine clinical care among adolescents experiencing high rates of VF.

## Methods

We conducted a randomized control trial (RCT) on adolescent and young adults (aged 10–24 years) who were failing ART comparing a peer support intervention plus standard of care (SOC) versus SOC alone from July 2016 to February 2018. The participants had been on antiretroviral medicines for at least 12 months and perinatal infection was presumed. The study was conducted at PHFCC, one of the largest referral hospitals in Zimbabwe. Study participants were screened and enrolled at PHFCC. Potential participants were also screened at four other adolescent clinics (i.e. Harare Hospital (another referral hospital), Wilkins Infectious Diseases Hospital and Beatrice Road Infectious Diseases Hospital in Harare). All study visits were conducted at PHFCC. Study participants resided within 50 km of Parirenyatwa Hospital, Harare Hospital, City of Harare Clinics, and other clinics within Harare.

The study participants had VF defined as at least two consecutive HIV viral load (VL) results of > 400 copies/mL more than 1 month apart or a recent VL of > 400 copies/mL in the past 12 months. Initial study enrollment was slower than anticipated due to challenges in recruiting participants who had not previously been exposed to the Zvandiri program as well as the need to limit clinic visits among adolescent students so that participation did not interfere with school attendance and exam schedules. Due to slow initial enrollment, eligibility criteria were amended to include participants who had at least one VL > 400 copies/mL in the previous 12 months to increase enrollment but only 9 participants were added to the final study sample using the amended VF criteria. The VL > 400 copies/mL threshold was selected as a conservative VL measurement as described in early studies on assessment of virological failure [24]. Patients who could not provide informed consent or assent and who had past or current involvement in the Zvandiri intervention program were excluded. Participants were randomly assigned in a 1:1 ratio into the intervention or SOC using a computer-generated randomization scheme with pre-prepared sequential randomization that was placed in sealed opaque envelopes by the study coordinator. Enrolled participants who met the inclusion criteria were given the opaque envelope which they opened themselves to reveal the arm they were assigned.

### Study procedures

All participants received their routine HIV care from the national program at no cost at PHFCC from medical staff who were not part of the study. Study procedures included a complete physical examination, evaluation of medical history and other baseline parameters at enrollment by research staff. Study related visits occurred at 12-week intervals (baseline, 12, 24, 36, and 48 weeks). The study results were reported up to week 36. Visits included VL testing (Roche Ampliprep Cobas v2.0 (Roche, USA)), CD4 count testing (Sysmex-Partec Cyflow^R^ Counter (CY-S-3022) (Muenster, Germany)), self-reported adherence assessments, and physical examination. The research nurses were not responsible for daily participant care, and referred participants to the clinical care providers, but they ensured study participants adhered to clinic appointments. Given that this was an implementation science study, providers were not given training on management of VF for the purposes of this study but were expected to apply the national guidelines as to when to switch ART as per SOC [25, 26]. The guidelines at that time relied on clinical measures, immunologic parameters and where available VL data. The study VL results were delivered to the clinic and results were relayed to the participants when they presented for care which was usually at the clinic every 2–3 months or earlier if they presented at an unscheduled visit. The study team did not determine when patients presented for these visits as this was determined by their primary caregiver at the clinic. The study team hypothesized that effects of the intervention would be seen within 48 weeks. However, the study was terminated once most participants had reached 36 weeks due to funding constraints. The study team ensured the study timeline visits occurred as scheduled at weeks 12, 24, and 36 and there were no stopping rules as it had been decided a priori that the study team would follow these participants for 48 weeks which was considered an adequate time to see the effects of the intervention.

The participants and their clinic staff were aware from the inception of this study that they would not get the VL results in real time and clinic staff were switching therapies mostly based on clinical failure. Clinical failure was defined as stages WHO-3 and WHO-4 for children and WHO-4 for both children and adults, but tuberculosis was excluded from determining clinical failure. A genotypic analysis was conducted on a subset of participants (n = 160) with VL ≥ 1000 copies/mL at enrollment. Viral ribonucleic acid (RNA) was extracted from plasma and genotyped by Sanger sequencing. Drug resistant mutations (DRM) were determined using the Stanford HIV database. The total genotypic susceptibility scores (tGSS) were calculated based on the number of ‘active’ drugs prescribed using the online Stanford HIV database genotypic resistance interpretation system [27].

### Standard of care (SOC)

All participants received SOC at the PHFCC as described in the “Guidelines for Antiretroviral Therapy for the Prevention and Treatment of HIV in Zimbabwe” [25, 26]. Trained primary care counselors (PCCs) conducted individual and group-level counseling as part of routine provision of medical care. The PCCs followed the standard clinic guidelines. In the group counseling sessions, the PCCs would discuss different topics that affect young people living with HIV such as adherence, disclosure, dating and practicing safe sex. In the individual sessions, the PCCs would focus on providing counselling on adherence and any other difficulties that the participant would be facing. Youth were encouraged to complete a self-reported adherence questionnaire about the number of missed doses over the previous 30 days and PCCs completed periodic pill counts. No interventions were targeted at caregivers. To avoid “contamination”, follow up clinic visits for the participants in the SOC arm were scheduled on different days from participants in the intervention arm.

### Intervention

Participants enrolled in the intervention arm received SOC as practiced at the PHFCC as well as ‘Zvandiri’ as the intervention. The Zvandiri intervention has been designed, evaluated and scaled over the last 18 years in response to the evolving clinical and psychosocial needs of young people growing up with HIV and is detailed elsewhere [15, 22]. “Zvandiri” is a peer-led model of layered psychosocial support services informed by the Unified Theory of Behaviour [21], delivered through home visits, support groups, clinic visits and mobile health, together with support for caregivers. On enrollment in the study, each participant was referred by the clinic to Africaid’s Zvandiri programme and assigned to a Community Adolescent Treatment Supporter (CATS) living within their own community. CATS are young people (18–24 years old) living with HIV who are recruited, trained, and mentored as peer counsellors by Africaid and the Ministry of Health and Child Care. Each participant received a package of layered services from their respective CATS throughout the duration of the study. CATS conducted weekly home visits for participants who had consented to be visited, during which they provided information, counselling, adherence monitoring and support. CATS were trained and mentored to utilize adolescent-sensitive counselling skills and techniques with participants to identify and address adherence barriers. If participants preferred not to be visited at home, they arranged to meet in an alternative venue considered to be safe and acceptable for the participant.

Each CATS delivered weekly WhatsApp messages to their designated participant. The messages provided adherence and clinic reminders and enquired about the participant's well-being. If participants did not have data but wanted to contact the CATS, they sent free ‘call me back’ messages to their CATS indicating they would like them to call. Not all participants had their own phone, and some opted to use their caregiver’s phone. Participants were invited to attend a monthly support group meeting facilitated by a professional HIV counselor together with a CATS at a community venue which participants considered to be safe, private, and accessible. The monthly support group addressed HIV, adherence, and coping strategies as well as issues of disclosure, relationships, sexual and reproductive health rights. Participants’ caregivers were also invited to attend a caregiver workshop that addressed communication and parenting skills, disclosure, HIV, ART, and adherence, to build skills to support adolescents. CATS were trained to identify and refer participants with ‘red flags’ indicating the need for further investigations and management such as ill health, psychological distress and protection risks. If participants were unwell or experienced psychosocial distress, they were referred by their CATS to Africaid’s multidisciplinary community outreach team (social worker, nurse, and counsellor) who then liaised with PHFCC. CATS did not provide any facility-based services although this is standard practice in Zvandiri-supported sites elsewhere.

### Primary outcome

The primary outcome of this trial was the proportion of participants who were virologically suppressed (defined as HIV VL of < 1000 copies/mL as per WHO guidelines adopted nationally and being used at that time) in the intervention group compared to SOC at week 36 [25].

### Statistical analyses

**Fig. 1:**
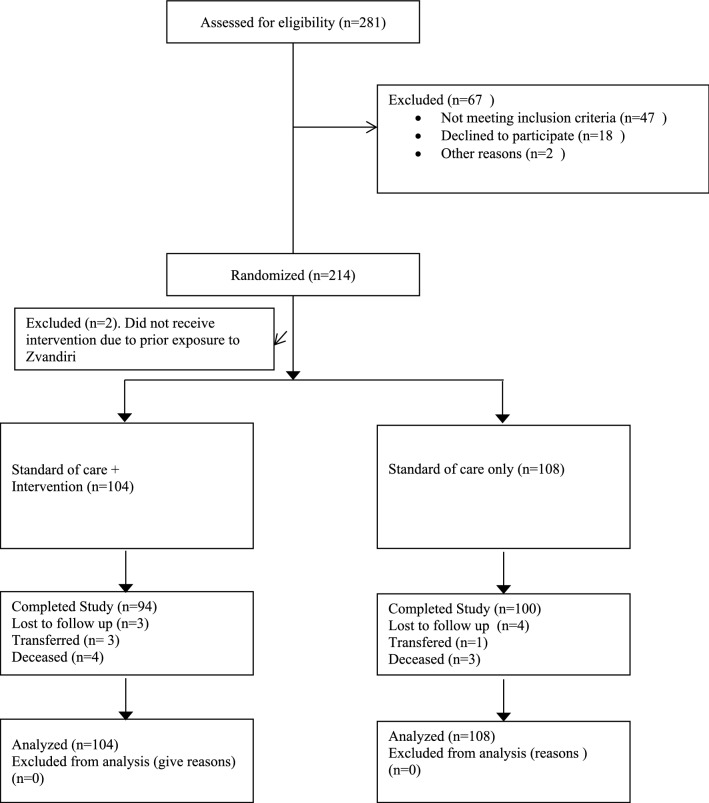
Consort diagram

The sample size for the study was determined by evaluating the difference between proportions of VF between the two groups based on a 33% observed treatment failure rate among adolescents. We assumed that the SOC group would have a failure rate of 25%, and that the Zvandiri intervention group would have a failure rate of 10%. Based on these assumptions, we determined that 250 participants enrolled in the study provided us with 80% power to detect a difference in efficacy between the two groups of 15%. The study screened 281 participants, and 214 were enrolled and randomized into the study groups. However, 2 participants who had been randomized into the intervention arm, were terminated early for protocol deviation (Fig. 1) and due to funding ending before all participants reached the 48 week mark, we conducted the analysis on participants enrolled and followed up for a total of at least 36 weeks with study closing in February 2018.

Medians and interquartile ranges (IQR) were computed for continuous variables and counts with percentages for categorical variables at baseline and for each time point. Chi-square statistics for categorical variables and two-sample rank sum (Mann–Whitney) for continuous variables were used to compare the intervention and SOC groups. We constructed a generalized estimating equation (GEE) model [28] with an exchangeable correlation structure with detectable VL as the outcome variable. We controlled for the follow-up period and study group. We also constructed an interaction variable between the study period and group.

We compared the virologic suppression rates between the two arms at 12 weeks, 24 weeks, and 36 weeks. Of the 212 participants, 47 missed at least one of the three follow-up visits including 33 participants at week 36. To ensure that the missing values did not reduce the precision of the estimates, we carried the last observation forward for missing time points assuming that the individual has not changed over time [29]. An intention to treat (ITT) analysis was conducted, followed by a subset analysis including only participants with an undetectable VL at baseline but viremia in the preceding 12-months. Data management and statistical analyses were performed using Stata/SE 14 for Windows (StataCorp LP, College Station, TX).

### Ethical approval

The study was approved by the Joint Research Ethics Committee (JREC) for the University of Zimbabwe College of Health Sciences and the Parirenyatwa Group of Hospitals (JREC 185/15), the Medical Research Council of Zimbabwe (MRCZ/A/1992), and the Massachusetts General Hospital Institutional Review Board (2016P000043). Participants provided informed consent if they were age 18 or older, those below the age of 18 provided assent and their guardians/parents provided parental informed consent.

## Results

### Baseline socio-demographics

A total of 212 participants were enrolled and followed up from June 2016 to February 2018 (n = 104 intervention and n = 108 SOC) (Table 1). The participants’ median age was 18.1 (IQR: 15.1–20.0) years. Half of the participants (50.5%, n = 107) were male. Nearly two-thirds of the participants (63.2%, n = 134) were adolescents i.e. aged 10–19 years. The majority (69.1%, n = 146) were students and 16.5% were unemployed. Among the participants, 17 (8.0%) reported being sexually active.

**Table 1:** Baseline socio-demographic characteristics of participants

	Total N = 212	Intervention N = 104	Standard of care N = 108
Male, n (%)	107 (50.5%)	50 (48.1%)	57 (52.8%)
Age, years median (IQR)	18.1 (15.1–20.0)	18.1 (14.7–20.1)	18.2 (15.7–19.9)
Age in years, n (%)			
10–14	40 (18.9%)	22 (21.2%)	18 (16.7%)
15–19	94 (44.3%)	45 (43.3%)	49 (45.4%)
20–24	78 (36.8%)	37 (35.6%)	41 (37.9%)
Sexually active, n (%)	17 (8.0%)	10 (9.6%)	7 (6.5%)
Employment Status: n (%)			
Employed	18 (8.5)	11 (10.6%)	7 (6.5%)
Unemployed	35 (16.5%)	17 (16.4%)	18 (16.7%)
Student	146 (69.1%)	70 (67.3%)	76 (70.4%)
Not attending school	13 (6.1%)	6 (5.8%)	7 (6.5%)
Participants 18 and older	N = 113	N = 60	N = 53
Participants 18 and older with educational level of secondary or above, n (%)	91 (80.5%)	42 (79.2%)	49 (81.6%)
Caregiver’s of participants under 18	N = 97	N = 48	N = 49
Caregivers of participants under the age of 18 with educational level of secondary education or above, n (%)	61 (62.9%)	29 (59.1%)	33 (66.7%)

### Baseline clinical characteristics

There were no clinical differences between the groups (Table 2). The participants had initiated ART as children likely due to perinatal infection and had been on ART with a median time of 6.4 (IQR: 3.8–8.5) years. Most participants were viremic, with 86.3% with a VL ≥ 1000 copies/mL; the median VL was 4.3 log_10_ copies/mL (IQR: 3.7–4.8). A large proportion of the participants had advanced immunosuppression with a median CD4 count of 217 cell/mm^3^ (IQR: 69–392), 47.2% had a CD4 count below 200 cells/mm^3^. Among participants for whom data on their ART regimens were available (n = 202), 142 participants (70.2%) had been on first line therapy i.e. non-nucleoside based therapy whilst 60 (29.7%) had been on second line therapy i.e. protease inhibitor (PI) based therapy. Most of the participants on first line therapy were on a once daily fixed dose combination consisting of Tenofovir/Lamivudine/Efavirenz (n = 93, 46.0%).

**Table 2:** Baseline clinical characteristics of enrolled participants

	Total N = 212	Intervention N = 104	Standard of care N = 108
CD4 count (cells/mm^3^) Median (IQR), (n =)	217 (69–392), (n = 208)	217 (95–409), (n = 101)	211 (52–381), (n = 107)
CD4 Count < 200 cells/mm^3^, n (%)	100 (47.2%)	48 (46.2%)	52 (48.2%)
HIV VL			
Viral load > 400 copies/mL, n (%)	192 (90.6%)	92 (88.5%)	100 (92.6%)
Viral load ≥ 1000copies/mL, n (%)	183 (86.3%)	89 (85.6%)	94 (87.0%)
Viral Load (log_10_ copies/mL) Median (IQR)	4.3 (3.7–4.8)	4.2 (3.6–4.7)	4.5 (3.8–4.9)
Duration on ART and self reported adherence			
Duration in years on ART Median (IQR), (n =)	6.4 (3.8–8.5), (n = 211)	6.5 (3.9–8.6), (n = 104)	6.4 (3.8–8.4), (n = 107)
Self-reported adherence (95% or higher), n (%)	Total N = 180 134 (74.4%)	61 (67.8%)	73 (81.1%)
ART Regimen	(N = 202)	(N = 101)	(N = 101)
First Line ART Regimen, n (%)	142 (70.2)	69 (68.3)	73 (72.2)
Tenofovir/Lamivudine/Efavirenz	93 (46.0%)	44 (43.6%)	49 (48.5%)
Tenofovir/Lamivudine/Nevirapine	11 (5.5%)	5 (5.0%)	6 (5.9%)
Zidovudine/Lamivudine/Efavirenz	11 (5.5%)	7 (6.9%)	4 (4.0%)
Zidovudine/Lamivudine/Nevirapine	24 (11.9%)	11 (10.9%)	13 (12.9%)
Stavudine/Lamivudine/Efavirenz	1 (0.5%)	1 (1.0%)	0 (0%)
Other	2 (1.0%)	1 (1.0%)	1 (1.0%)
Second Line ART Regimen, n (%)	60 (29.7%)	32 (31.7%)	28 (27.7%)
Tenofovir/Lamivudine/Atazanavir/Ritonavir	33 (16.3%)	17 (16.8%)	16 (15.8%)
Zidovudine/Lamivudine/Atazanavir/Ritonavir	9 (4.5%)	6 (5.9%)	3 (3.0%)
Zidovudine/Lamivudine/Lopinavir/Ritonavir	2 (1.0%)	0 (0%)	2 (2.0%)
Abacavir/Lamivudine/Atazanavir/Ritonavir	9 (4.5%)	4 (4.0%)	5 (5.0%)
Abacavir/Lamivudine/Lopinavir/Ritonavir	7 (4.0%)	5 (5.0%)	2 (2.0%)

Using the WHO criteria for VF, at baseline, the VL was ≥ 1000 copies/mL in 89 participants (85.6%) in the intervention arm and 94 (87.0%) in the SOC arm. At week 12, the proportion of subjects with a VL ≥ 1000 copies/mL, was lower in the intervention arm than in the SOC (88.5%, (n = 92) vs. 93.5% (n = 101), p = 0.198), however this difference was not statistically significant (Table 3). At week 24, the proportion of subjects with a detectable viremia was significantly lower in the intervention arm than in the SOC arm (76.0% (n = 79) vs. 89.0% (n = 96), p = 0.013). At week 36, there remained a difference in the proportion of subjects with a detectable VL between the intervention arm (68.3%, n = 71) and SOC arm (79.6%, n = 86), which was marginally statistically significant (p = 0.059).

**Table 3:** Clinical characteristics over time

	Time point	Total N (%)	Intervention N (%)	Standard of care N (%)	p-value*
Viral load (log_10_ copies/ml) Median(IQR)	Baseline	4.3 (3.7–4.8)	4.2 (3.6–4.7)	4.5 (3.8–4.9)	
	Week 12	3.9 (2.2–4.6)	3.6 (2.0–4.4)	4.1 (2.7–4.7)	0.041*
	Week 24	3.6 (2.0–4.5)	3.2 (1.5–4.5)	3.8 (2.3–4.5)	0.111
	Week 36	3.1 (0.0–4.6)	2.7 (0–4.5)	3.4 (1.7–4.6)	0.113
Viral load ≥ 1000 copies/mL n (%)	Baseline	200 (94.3%)	183 (86.3%)	89 (85.6%)	
	Week 12	193 (91.0%)	92 (88.5%)	101 (93.5%)	0.198
	Week 24	175 (82.6%)	79 (76.0%)	96 (89.0%)	0.013*
	Week 36	157 (74.1%)	71 (68.3%)	86 (79.6%)	0.059
Self-reported adherence (95% or higher), n (%)	Baseline	134 (74.4%)	61 (67.8%)	73 (81.1%)	
	Week 12	144 (68.3%)	70 (67.3%)	74 (69.2%)	0.773
	Week 24	140 (68.0%)	68 (66.7%)	72 (69.2%)	0.693
	Week 36	139 (67.5%)	68 (66.0%)	71 (68.9%)	0.655

^*^Statistical comparison is intervention compared to standard of care

The median VL was noted to be lower in the intervention group at week 12; 3.6 log_10_ copies/mL (IQR 2.0–4.4) vs 4.1 log_10_ copies/mL (IQR 2.7–4.7), p = 0.041 (Table 3). The proportion that showed viral suppression at week 36 was poor in both groups i.e. 31.7% (intervention) vs 20.4% (SOC). We evaluated the factors associated with a detectable VL. Table 4 presents a GEE model that tests whether participants in the intervention group were significantly less likely to have a detectable VL after controlling for follow up time, enrollment arm, and the interaction term. There was no difference in the probability of having a detectable VL in the intervention group compared to the SOC group (odds ratio (OR) 1.14 (95% confidence interval (CI): 0.82–1.59), p-value = 0.439).

**Table 4:** Results of GEE model on detectable viral load

Independent variables	Odds ratio (95% confidence intervals (CI))	p-value
Intervention vs sandard of care model		
Intervention	0.36 [0.12–1.03]	0.058
Time	0.50 [0.38–0.65]	0.000***
Intervention*Time interaction	1.14 [0.82–1.59]	0.439

***p < 0.001

Genotyping was conducted on stored baseline plasma samples from 185 participants (87%) with VL ≥ 1000 copies/mL at the time of study enrollment. Of the 185 participants, 160 (86%) were successfully genotyped with 112 (70%) on 1st line ART regimen and 48 (30%) on 2nd line PI based ART. High levels of Non-nucleoside reverse transcriptase inhibitor (NNRTI) DRM were observed in 44% of participants in the intervention group and 56% in the SOC group (p = 0.146). PI associated mutations were detected in only 10% of 2nd line recipients (Additional file 1: Table S1). Stanford inferred drug resistance scores were used to calculate the tGSS to the baseline regimen. There was no significant difference in tGSS in the two groups, however the majority 110 (68.7%) had tGSS ≤ 2 to their baseline regimen.

We evaluated individuals who switched during the study, the time to switch and barriers to switching therapy. Genotyping was not available as routine clinical care. The clinical care providers (not the study researchers) made decisions on treatment switches within the context of routine clinical care and did not receive VL results in real time. Among the 212 study participants, only 70 (33%) participants switched to a new regimen during the study—31 in intervention arm and 39 in the SOC care (p = 0.24). These 70 participants had a lower median CD4 count at baseline compared to those who did not switch, although not statistically significant [172 (45–338) vs 235 (100–428), p = 0.072]. Most of the participants who switched, 86% (n = 62/70) were first line failures. These 62 participants switched to a PI based 2nd line ART regimen. Although only 33% (n = 70) of the participants switched to a new ART regimen, the “blind” switching i.e. switching using clinical criteria and not VL results by the health caregiver made a difference. After switching, a statistically significant difference in median (IQR) VL at week 36 was observed in those who were switched compared to those who did not switch [1.86 (1.3–4.0) vs 3.66 (2.17–4.72), p = 0.0001]. The number of regimen switches was not sufficiently large to evaluate the interaction between regimen switch and study groups (intervention vs. SOC). We observed a clinically significant difference in the median VL among those who switched regimens during the course of the study [from median (IQR) VL of 4.5 (4.1–4.9) to median (IQR) VL of 1.86 (1.3–4.0) respectively]. See Additional file 1: Table S2. Genotypic analysis of a subset of participant baseline samples (n = 160) suggested that this lack of VL suppression despite the “complex” intervention was due to high rates (86%, n = 137/160) of drug resistance in the whole study sample.

Providers did not make treatment switches in accordance with national guidelines. The main reasons for not switching included concerns about poor adherence using existing regimens (80%), waiting for genotyping results if the participant was on second line therapy (8%), history of ART defaulting (7%), observed susceptibility to virus when genotype results were eventually available (3%) and mental health concerns (2%) (e.g. suicidality). The concerns about poor adherence were supported by the participants low rates of self-reported adherence (Table 3).

## Discussion

This study aimed to determine the effectiveness of a community-based peer support intervention compared to the SOC by measuring adherence and virologic suppression rates at 12, 24, 36 and 48 weeks among 250 adolescents and youth (10–24 years) who were failing their ART regimens. However, only 212 participants were enrolled and followed up for 36 weeks due to slow study enrolment as well as ending of the grant. The study found a transient effect of the Zvandiri peer intervention in improving virological suppression among adolescents and young adults failing ART. However, the overall rates of viral suppression rates were poor in both groups (i.e. 31.7% in the intervention group vs 20.4% in the SOC) and high levels of NNRTI DRM were observed in both arms at baseline.

The benefits of integrated peer-led, community interventions within adolescent HIV care and treatment at primary health care clinics have had an effect on linkage and retention in care, adherence and psychosocial well-being in Zimbabwe [16]. Yet evidence of their effect in achieving viral suppression among adolescents failing treatment at a tertiary level health care clinic had been limited. Our participants were most likely infected during the perinatal period as suggested by the 1:1 male to female ratio, the early age at ART initiation, long median duration on ART (6 years) and the low rate (8%) of self-reported sexual activity. Adolescence is a period which is also well known to be associated with ART adherence challenges [30–33].

Although, the viral suppression rates achieved during the study were low in both study groups, we found a statistically significant difference in the proportion of participants with a detectable viremia in the intervention arm than in the SOC arm (76.0% (n = 79) vs 89.0% (n = 96), p = 0.013) at week 24. The same observation was seen at week 36, however with a marginally statistically significant difference (p = 0.059). Failure to achieve statistical significance may be due to the sample sizes getting smaller per group over time. After switching ART regimens, a statistically significant difference in median (IQR) VL at week 36 was observed in those who were switched compared to those who did not switch [1.86 (1.3–4.0) vs 3.66 (2.17–4.72), p = 0.0001]. As expected, those who were switched by their clinicians were more likely to have been on a 1st line regimen (88.6%) at baseline. This was an expected finding as the national guidelines allowed for such patients to be switched to the 2nd line regimen without VL testing. This 2nd line regimen was explicitly stated in the national guidelines and would have entailed changing the nucleoside backbone, retaining lamivudine but including a PI. Less (58.4%) of those who did not switch regimens were on 1st line regimens at baseline. This group is likely to have had those already using a PI i.e. presumed to be on 2nd line therapy and thus needed more laboratory testing such as VL and genotyping to switch to the next regimen i.e. 3rd line ART. Hence, the intervention group played a significant role in improving virological suppression among this population (31.7% vs 20.4%).

Generally, the healthcare delivery system allowed for delayed switching by primary providers as there was no real time access to VL results and no access to genotyping results. Additionally, the clinic staff concerns for low adherence matched the low self-reported adherence rates greater than or equal to 95% (i.e. intervention arm 68% vs SOC arm 81%). Thus, the providers may have been reluctant to switch given the limited treatment options, including need for HIV genotyping prior to switch, cost issues as well as concerns about the high pill burden of the available 3rd line medicines. According to the Zimbabwean national ART guidelines (2016), all HIV patients were not switched to the next regimen, in particular the second line therapy, whilst attempts were made to provide “enhanced adherence counselling” which would occur over at least 3 months. Thus, the clinic providers did not have sufficient objective information to inform the decision to switch at the time that this study was conducted. Viral load testing is now more accessible, and it is likely that switches are being implemented much earlier than before. The national guidelines now allow for two VL tests during the first year at 6 months apart and thereafter annually. Our findings confirmed the need for objective laboratory testing i.e. frequent VL testing, as opposed to depending on clinical failure in these adolescents living with HIV whom we already know have difficulty with adhering to their therapies. Use of clinical monitoring in adolescents and youth will be costly to the nation in the long run.

The intervention consisted of weekly home visits, daily WhatsApp messages, monthly support group meetings and caregiver meetings. The SOC participants did not attend the support group format but attended group counseling visits that may have provided similar social interactions at PHFCC. The GEE model conducted in this study seems to suggest that attending (as per intervention arm) support groups, receiving home visits and the daily text messages did not increase the probability of having an undetectable VL at week 36. However, the GEE model did not control for social and environmental factors including mental health conditions that have been shown to influence a participant’s behavior and interfere with the efficacy of a psychosocial intervention [12–14]. This study found that the adolescent and young adult cohort with VF did not suppress, despite enhanced support from peers in the intervention arm. This may be due to high rates of DRM observed at baseline. Long term ART e.g. median ART duration 6.4 years as was found in our study population with chronic and/or intermittent periods of low treatment adherence would be expected to result in the emergence of DRMs. A recent study in Zimbabwe found high levels (97%) of DRMs among adolescents and young adults failing first-line ART, despite enhanced adherence counselling [27]. High levels of drug resistance have been noted among adolescents and young adults in other studies [25–28]. Further analysis is also needed to ascertain the role that poor mental health may have had on adherence and viral suppression in this population, considering that adolescents living with HIV are at risk of mental health conditions correlating with poor adherence.

Several study limitations may influence these findings. It was difficult to blind participants and the researchers against knowing which participant was in which group. Attempts were made to keep the groups separate by having the different groups attending clinics on different days but it is possible that some cross-contamination could have occurred in the community where participants could have shared their care. In addition, study participants may have shared their experiences with peer supporters resulting in the possibility of cross-contamination between study groups. We amended the protocol to include participants with only one detectable VL result due to slow enrollment and approximately 25% of study participants missed a follow-up visit including 15% at week 36 resulting in the need to impute values for a subset of the analyses. The study was limited in its ability to evaluate the interaction between ART regimen switch and study group due to the relatively low numbers of participants that were switched i.e. 70 out of the 212 enrolled in this study. Another limitation includes the lack of drug resistance data at the end of the study (week 36) as a proxy to infer adherence to the regimens among the failures.

We conclude that improving viral outcomes in this population will require an approach that combines psychosocial support to promote adherence, coupled with drug resistance and VL data that are made available to primary providers in a timely manner to support drug switching and ongoing adherence support. In addition, more robust treatment regimens with higher barriers to resistance are required for this heavily treatment experienced populations with significant resistance. Improving access to diagnostic data in particular drug resistant and VL monitoring data throughout the course of the study as well as providing training and support to practitioners caring for chronically infected adolescents and youth will be important to achieve better outcomes for this population.

## Supplementary Information

**Additional file 1: Table S1.**Baseline Genotyping on participants by arm. **Table S2.** Clinical and demographic characteristics of subjects who switched and did not switch. **Table S3.** Exposure to home visits, daily sms and support group attendance over time among participants enrolled in the intervention.

## Data Availability

All data relevant to this publication can be obtained by request to the authors.

## Abbreviations

ART: Antiretroviral therapy
CATS: Community Adolescent Treatment Supporter
CI: Confidence interval
DRM: Drug resistant mutations
GEE: Generalized estimating equation
HIVDR: HIV drug resistance
IQR: Interquartile range
ITT: Intention to treat
JREC: Joint Research Ethics Committee
MRCZ: Medical Research Council of Zimbabwe
NNRTI: Non-nucleoside reverse transcriptase inhibitor
OR: Odds ratio
PCC: Primary Care Counselors
PHFCC: Parirenyatwa Hospital Family Care Clinic
PI: Protease inhibitor
RCT: Randomized control trial
RNA: Ribonucleic acid
SOC: Standard of care
tGSS: Total genotypic susceptibility scores
VF: Virologic failure
VL: Viral load
