# Supplementary material for: The transient effect of a peer support intervention to improve adherence among adolescents and young adults failing antiretroviral therapy in Harare, Zimbabwe: a randomized control trial
Source: AIDS Res Ther. 2021 Jun 16;18:32. doi: 10.1186/s12981-021-00356-w (PMC8207727; doi:10.1186/s12981-021-00356-w)
Supplement: Supplementary file 1 — Additional file 1: Table S1.Baseline Genotyping on participants by arm. Table S2. Clinical and demographic characteristics of subjects who switched and did not switch. Table S3. Exposure to home visits, daily sms and support group attendance over time among participants enrolled in the intervention. [file 12981_2021_356_MOESM1_ESM.docx]

**Additional file**

**Table S1: Baseline Genotyping on participants by arm**

| ***Variables*** | ***Intervention*** | ***Standard of Care*** | ***p value*** |
| --- | --- | --- | --- |
| Drug Resistant Mutations (DRMs) observed (n=137) | 60 (44%) | 77 (56%) | 0.146 |
| NRTI associated mutations (n=96) | 46 (48%) | 50 (52%) | 0.683 |
| NNRTI associated mutations (n=137) | 60 (44%) | 77 (56%) | 0.146 |
| PI associated mutations (n=5) | 1 (20%) | 4 (80%) | NA |
| **Total Genotypic Susceptibility Scores (tGSS) to baseline regimen:** | | | |
| tGSS≤2 (n=110) | 46 (42%) | 64 (58%) | 0.086 |
| tGSS>2 (n=50) | 27 (54%) | 23 (46%) | 0.572 |

**Table S2. Clinical and demographic characteristics of subjects who switched and did not switch**

| Participants (n=160) | Switch (n=70) | No switch (n=142) | p |
| --- | --- | --- | --- |
| **Gender**  **Male, n (%)** | 35 (50%) | 71 (50%) |  |
| **Age in years, Median(IQR)** | 18 (16-19) | 18 (14-20) | 0.66 |
| **Duration on ART, median years (IQR)** | 6.23 (3.79-8.4) | 6.52 (3.9-8.6) | 0.54 |
| **Baseline CD4 Count (cells/mm^3^) (Median/IQR)** | 172 (45-338) | 235 (100-428) | 0.072 |
| **Viral Load BL (log_10_ copies/mL) (Median/IQR),** | 4.5 (4.1-4.9)  (n=70) | 4.2 (3.39-4.75)  (n=137) | 0.0048 |
| **Viral Load W36 (log_10_ copies/mL) (Median/IQR)** | 1.86 (1.3-4.0)  (n=57) | 3.66 (2.17-4.72)  (n=103) | 0.0001 |
| **First line regimen at baseline (n=145)** | 62 (88.6%) | 83 (58.4%) | <0.0001 |

**Table S3. Exposure to home visits, daily sms and support group attendance over time among participants enrolled in the intervention**

|  | **Total exposure day 1-week 36 (Median/IQR)** | **From Day 1-Week 12 (Median/IQR)** | **From Week 12- Week 24 (Median/IQR)** | **From Week 24-36 (Median/IQR)** |
| --- | --- | --- | --- | --- |
| Received home visits (all) | 11  (IQR 8-12) | 8  (IQR 7-11) | 12  (IQR 10-13) | 11  (IQR 9-13) |
| Received home visits (no detectable viral load) | 12  (IQR 10-13) | 9.5  (IQR 7-11) | 12  (IQR 10-12) | 12  (IQR 10-13) |
| Received home visits (detectable viral load) | 10  (IQR 8-12) | 8  (IQR 7-11) | 12  (IQR 9-14) | 11  (IQR 8-13) |
| Exposure to daily sms (all) | 61  (IQR 32-86) | 63  (IQR 52-77) | 78  (IQR 36-89) | 36  (IQR 10-76) |
| Exposure to daily sms (no detectable viral load) | 63  (IQR 23-89) | 66  (IQR 37.5-87) | 86  (IQR 42-92) | 42  (IQR 16-87) |
| Exposure to daily sms (detectable viral load) | 60  (IQR 36-85) | 63  (IQR 53-77) | 78  (IQR 36-89) | 26  (IQR 9-63) |
| Support group attendance (all) | 3  (IQR 2-6) | 2  (IQR 1-4) | 4  (IQR 2-6) | 3  (IQR 2-6) |
| Support group attendance (no detectable viral load) | 4  (IQR 2-6) | 2  (IQR 1.5-3) | 4  (IQR 3-6) | 4  (IQR 2-6) |
| Support group attendance (detectable viral load) | 3  (IQR 2-6) | 2  (IQR 1-4) | 4  (IQR 2-6) | 3  (IQR 1-6) |
